# IL-12/15/18-induced cell death and mitochondrial dynamics of human NK cells

**DOI:** 10.3389/fimmu.2023.1211839

**Published:** 2023-07-27

**Authors:** Iñigo Terrén, Víctor Sandá, Ainhoa Amarilla-Irusta, Ainara Lopez-Pardo, Arrate Sevilla, Gabirel Astarloa-Pando, Laura Amo, Olatz Zenarruzabeitia, Luca Scorrano, Francisco Borrego

**Affiliations:** ^1^ Immunopathology Group, Biocruces Bizkaia Health Research Institute, Barakaldo, Spain; ^2^ Department of Genetics, Physical Anthropology and Animal Physiology, University of the Basque Country, Leioa, Spain; ^3^ Ikerbasque, Basque Foundation for Science, Bilbao, Spain; ^4^ Department of Biology, University of Padova, Padova, Italy; ^5^ Veneto Institute of Molecular Medicine, Padova, Italy

**Keywords:** NK cells, mitochondria, cytokine-preactivated NK cells, memory-like, cancer immunotherapy, IL-12, IL-15, IL-18

## Abstract

Natural killer (NK) cells are lymphocytes with potent antitumor functions and, consequently, several NK cell-based strategies have been developed for cancer immunotherapy. A remarkable therapeutic approach is the adoptive transfer of NK cells stimulated with IL-12, IL-15 and IL-18. This cytokine stimulation endows NK cells with properties that resemble immunological memory and, for this reason, they are known as cytokine-induced memory-like (CIML) NK cells. Very promising results have been reported in clinical trials and yet, there are still unknown aspects of CIML NK cells. Here, we have conducted a preliminary study of their mitochondrial dynamics. Our results show that upon IL-12/15/18 stimulation the viability of NK cells decreased and an increment in mitochondrial superoxide levels was observed. In addition, we found that mitochondria appeared slightly elongated and their cristae density decreased following IL-12/15/18 stimulation, possibly in a process mediated by the low levels of optic atrophy type 1 (OPA1) protein. Interestingly, although mitophagy was slightly impaired, an increase in autophagic flux was observed, which might explain the reduced viability and the accumulation of unfit mitochondria. Our findings could be of relevance in order to design new strategies intended to improve the mitochondrial fitness of IL-12/15/18-stimulated NK cells with the aim of improving their therapeutic efficacy.

## Introduction

Mitochondria are dynamic organelles that coordinate several cellular functions, including metabolism, differentiation, cell death and autophagy, among others (1). The relevance of mitochondria for natural killer (NK) cell effector functions has been evidenced, especially in the context of cancer. Zheng et al. reported that tumor-infiltrating NK cells from patients with liver cancer showed fragmented mitochondria and the upregulation of mitochondrial fission-related genes, lower mitochondrial membrane potential (Δψm), and increased mitochondrial reactive oxygen species (mtROS) levels were also found. They demonstrated how mitochondrial fragmentation reduced NK cell survival and how inhibiting mitochondrial fission improved their antitumor functions (2). Other authors have also reported evidences of mitochondrial dysfunction in NK cells from patients with colorectal liver metastasis (3), ovarian cancer (4), metastatic breast cancer (5), hepatocellular carcinoma (6) and neuroblastoma (7). Accumulation of dysfunctional mitochondria can cause oxidative stress, including an increased production of ROS, and induce cell death. Indeed, autophagic clearance of dysfunctional mitochondria is essential for the survival of adaptive long-lived NK cells (8). These studies highlight the idea that mitochondrial fitness is linked to NK cell effector functions in cancer, a hypothesis that has been demonstrated in other contexts as well (9, 10).

The adoptive transfer of interleukin (IL)-12/15/18-stimulated NK cells has emerged as a promising immunotherapy for different malignancies (11–16). Yet, the relevance of mitochondrial fitness for the survival and effector functions of IL-12/15/18-stimulated NK cells (also known as cytokine-induced memory-like or CIML) is mostly unknown. Therefore, new means to improve the efficacy of these therapeutic strategies are needed, and understanding the cytokine-induced changes in NK cells may represent such a mean. Here, we have studied the effect of IL-12/15/18 stimulation on the mitochondrial dynamics of human NK cells. We focused on the effect of cytokine stimulation on NK cell viability and its potential relationship with mitochondria and mitochondria-related processes. More specifically, we have analyzed mitochondrial quality through different parameters such as mtROS accumulation, Δψm, ultrastructural morphology, and the expression of relevant proteins for the mitochondrial architecture such as dynamin-related protein optic atrophy protein 1 (OPA1). Moreover, we have measured cellular processes involved in the quality control of mitochondria, including autophagy and mitophagy.

## Materials and methods

### Samples and cell culture

Buffy coats from healthy donors were provided by the Basque Biobank and the Immunotrasfusionale Unit from the University Hospital of Padova. All subjects provided written and signed informed consent in accordance with the Declaration of Helsinki. Fresh peripheral blood mononuclear cells (PBMCs) were obtained from buffy coats by Ficoll Paque Plus (GE Healthcare) density gradient centrifugation. NK cells were isolated from PBMCs by negative depletion with human NK cell Isolation Kit (Miltenyi Biotec). All the experiments were performed with purified NK cells (>80% CD3-CD14-CD19-CD56+ cells). Cells were cultured in RPMI 1640 medium supplemented with GlutaMAX (Gibco), 10% heat-inactivated FBS (HyClone), 1% non-essential amino acids (Gibco), 1% sodium pyruvate (Gibco) and 1% penicillin/streptomycin (Gibco), hereinafter, complete RPMI or cRPMI. In some experiments, serum-free NK MACs (Miltenyi Biotec) media was used instead. Purified NK cells were plated at 2×10^6^ cell/mL in 24-well plates and cultured at 37°C in the appropriate media for 16–18 hours with 10 ng/mL rhIL-12 (Miltenyi Biotec), 100 ng/mL rhIL-15 (Miltenyi Biotec) and 50 ng/mL rhIL-18 (MBL International Corporation), or cultured in media alone for the same time. Cells were then washed three times with PBS, plated at 2×10^6^ cell/mL in 24-well plates and cultured at 37°C in cRPMI for seven days with 1 ng/mL rhIL-15 or 20 U/mL rhIL-2 (Miltenyi Biotec). After 4 days, media and cytokines (rhIL-15 or rhIL-2) were replaced, and cells were cultured for 3 more days. The time-points after the 16-18 hour stimulation, and after seven days of culture, are referred to as Day 0 and Day 7, respectively (**Supplementary Figure S1**).

### Flow cytometry

For flow cytometry analyses, 10^6^ cells were collected in flow cytometry tubes and washed two times with PBS. Dead cells were identified by staining cells for 30 minutes on ice, or 20 minutes at room temperature (RT), with LIVE/DEAD Fixable Near-IR or Fixable Violet Dead Cell Stain Kit (Invitrogen) prior to extracellular staining. Apoptosis was measured by staining cells for 20 minutes at RT with 0.4 µM Apotracker Green (BioLegend), a phosphatidylserine-binding fluorescent probe. LIVE/DEAD and Apotracker dyes were used to discriminate between live (LIVE/DEAD-Apotracker-), early apoptotic (LIVE/DEAD-Apotracker+) and late apoptotic (LIVE/DEAD+Apotracker+) cells.

Extracellular staining was performed by washing cells two times with PBS containing 2.5% BSA (Sigma-Aldrich), and then incubating them for 30 minutes on ice with the following fluorochrome-conjugated mouse anti-human antibodies: BV421 anti-CD56 (NCAM16.2), BV510 anti-CD3 (UCHT1), BV510 anti-CD14 (MφP9), BV510 anti-CD19 (SJ25C1), and BV786 anti-CD56 (NCAM16.2) from BD Biosciences. Cells were then washed two times with PBS.

Mitochondrial ROS (mtROS) levels were measured by staining cells with 5 µM MitoSOX Red (Invitrogen) for 20 minutes at 37°C. Of note, mtROS levels were normalized to mitochondrial content, measured with MitoTracker. Mitochondrial mass was measured by staining cells with 150 nM MitoTracker Deep Red (MTDR) (Invitrogen) for 25 minutes at 37°C, or with 50 nM MitoTracker Green (MTG) (Invitrogen) for 30 minutes at 37°C. ΔΨm was measured by staining cells with 25 nM tetramethylrhodamine methyl ester (TMRM) (Invitrogen) for 30 minutes at 37°C. As a positive control to measure ΔΨm, cells were cultured with 2 µM oligomycin (Sigma-Aldrich) during TMRM staining. As a negative control to measure ΔΨm, cells were incubated with 2 µM FCCP (Sigma-Aldrich) for 2 minutes at RT before acquisition in the flow cytometer. To determine mitophagy, freshly isolated NK cells were transfected with a plasmid encoding mKeima-Red-Mito-7 (Addgene plasmid #56018, a gift from Michael Davidson) (17), hereinafter referred to as mKeima, and stimulated with IL-12/15/18 for 16-18 hours in the presence of 10 µM CCCP (Sigma-Aldrich) or an equivalent volume of vehicle control (DMSO). mKeima is resistant to lysosomal degradation and its fluorescence is pH-dependent. The emission wavelength of mKeima (~620 nm) is not modified at different pHs, but, when mitochondria are delivered from a neutral pH to the acidic pH of lysosomal environment, the peak of excitation spectrum shifts from 440 to 586 nm. Mitophagy inductor CCCP was used as a positive control to establish electronic gates discriminating between mKeima-expressing cells in acidic or neutral pH.

After the staining protocols, cells were washed two times with PBS containing 2.5% BSA and acquired in a LSRFortessa X-20 (BD Biosciences), FACSCanto II (BD Biosciences) or FACSAria IIIu (BD Biosciences) flow cytometers. NK cells were gated within viable lymphocytes as CD3-CD14-CD19-CD56+ cells. Flow cytometry data were analyzed with FlowJo v10.0.7 software (BD Biosciences).

### Cytochrome c release assay

Immunofluorescence staining of purified NK cells was performed to analyze cytochrome c release. Briefly, 13 mm round glass coverslips were plated in 24-well plates and washed with 70% ethanol and sterile distilled water. Once dried, 300 µL of filter-sterilized 0.01% poly-L-lysine (Sigma-Aldrich) were added to each well, and coverslips were incubated for 5 minutes at RT before washing them with sterile distilled water. Purified NK cells were washed and resuspended in sterile PBS after cytokine stimulation, and 10^6^ NK cells were added to each well. Plates were pulse-centrifuged at 200 g to facilitate NK cell adhesion to the poly-L-lysine-coated coverslips. As a positive control, cells were cultured with 1 mM hydrogen peroxide (Sigma-Aldrich) for 90 minutes at 37°C. Coverslips were then washed with PBS and cells were fixed by incubating them with 3.7% formaldehyde for 30 minutes at RT. Next, coverslips were washed with PBS and cells were permeabilized by incubating them with 0.1% Triton X-100 (Sigma-Aldrich) for 10 minutes at RT. Then, coverslips were washed with PBS and cells were blocked by incubating them with PBS containing 5% normal goat serum (Sigma-Aldrich) and 1% BSA for 20 minutes at RT. Coverslips were washed with PBS and then, cells were incubated with mouse anti-cytochrome c (6H2.B4, 1:200, BD Biosciences) and rabbit anti-TOM20 (polyclonal, 1:200, Proteintech) overnight at 4°C. Next day, coverslips were washed with PBS and cells were stained by incubating them with secondary antibodies AF488-labeled goat anti-mouse (polyclonal, 1:250, Invitrogen) and AF568-labeled donkey anti-rabbit (polyclonal, 1:250, Invitrogen) for 1 hour at RT. Coverslips were washed with PBS and distilled water and mounted on microscope slides with ProLong Gold Antifade mounting solution with DAPI (Invitrogen). Slides were analyzed with a ZEISS LSM 700 laser scanning confocal microscope, ZEISS Plan-Apochromat 63x/1.4 Oil DIC M27 objective and excited using the appropriate laser lines. Images were acquired using a 1024 × 1024 resolution with the ZEN microscopy software (ZEISS) and analyzed with Fiji software.

### Protein extraction and immunoblotting

Proteins were extracted from purified NK cells by incubating them for 30 minutes on ice with radioimmunoprecipitation assay (RIPA) buffer containing protease inhibitors cocktail (Sigma-Aldrich), followed by centrifugation at 15,000 rpm for 30 minutes at 4°C. Pellet was discarded and supernatant was stored at -20°C. Protein concentration was calculated by incubating samples with Pierce BCA Protein Assay Kit (Thermo Fisher Scientific) for 30 minutes at 37°C and measuring light absorbance at 562 nm with Varioskan LUX microplate reader (Thermo Fisher Scientific). To measure autophagic flux, NK cells were cultured with 0.1 mg/mL chloroquine (Sigma), or an equivalent volume of vehicle control (distilled water), for 2 hours at 37°C prior to protein extraction. Extracted proteins (15-20 µg) were mixed with NuPAGE LDS Sample Buffer (Invitrogen) containing 10% β-mercaptoethanol (Sigma-Aldrich) and heated at 95°C for 5 minutes. Proteins were then separated in homemade 15% SDS/PAGE gels or in NuPAGE 4 to 12% Bis-Tris gels (Invitrogen) with Tris-Glycine-SDS (Bio-Rad) or Tris-MOPS-SDS (GenScript) running buffers, respectively, and transferred to polyvinylidene fluoride membranes with 0.45 µm pore size (Merck). Proteins were blocked by incubating membranes with Tris-buffered saline containing 0.1% TWEEN 20 (Sigma-Aldrich) and 5% skim milk (Sigma-Aldrich) for 1 hour at RT. Proteins were then labelled by incubating the membranes overnight at 4°C with the following primary mouse or rabbit antibodies: anti-LC3B (polyclonal, 1:1000, Sigma-Aldrich), anti-vinculin (hVIN-1, 1:5000, Sigma-Aldrich), anti-OPA1 (18/OPA1, 1:1500, BD Biosciences), and anti-TOM40 (Santa Cruz Biotechnology, clone D-2). Membranes were washed for 30 minutes with Tris-buffered saline containing 0.1% TWEEN 20 and incubated for 1 hour at RT with the corresponding goat anti-mouse or goat anti-rabbit horseradish peroxidase-conjugated antibodies (1:3000, LI-COR). To study autophagic activity, we first determined LC3-II expression (normalized to vinculin) of control and activated NK cells. Then, autophagic flux was calculated by subtracting the LC3-II expression in cells exposed to vehicle control (distilled water) from the LC3-II expression in cells exposed to chloroquine. When required, antibodies were removed by incubating the membranes with WesternSure ECL Stripping Buffer (LI-COR) for 10 minutes at RT. After that, membranes were washed and blocked again as previously described. Chemiluminescence was measured in Alliance Q9 Atom imaging platform. Densitometric quantification was performed with Fiji software.

### Transmission electron microscopy

Purified NK cells were fixed by incubating them with 2.5% glutaraldehyde (Sigma-Aldrich) in 0.1M sodium cacodylate buffer (Sigma-Aldrich) pH 7.4 for 1 hour at 4°C. The samples were postfixed with 1% osmium tetroxide plus potassium ferrocyanide 1% in 0.1M sodium cacodylate buffer for 1 hour at 4°C. After three washes with distilled water, samples were dehydrated in a graded ethanol series and embedded in an epoxy resin (Sigma-Aldrich). Ultrathin sections (60-70 nm) were obtained with an Ultratome Leica Ultracut EM UC7 ultramicrotome, counterstained with uranyl acetate and lead citrate and viewed with a Tecnai G2 (FEI) transmission electron microscope operating at 100 kV. Images were captured with a Veleta (Olympus Soft Imaging System) digital camera and analyzed with Fiji software.

### NK cell transfection

Freshly isolated human NK cells were transfected with mKeima plasmid, encoding mKeima Red linked to mitochondrial targeting sequence from subunit VIII of human cytochrome c oxidase, and kanamycin and neomycin resistance genes. The plasmid was purified from competent *Escherichia coli* (DH5α) cells using EndoFree Plasmid Maxi Kit (QIAGEN) following manufacturer’ instructions and resuspended in endotoxin-free water. Plasmid concentration and quality of plasmid purification were assessed by measuring light absorption at 260 nm and calculating the ratio of light absorption at 260/280 and 260/230 nm with NanoDrop 2000 spectrophotometer (Thermo Fisher Scientific). Transfection was performed with Neon Transfection System (Thermo Fisher Scientific) following an optimized protocol. Briefly, 4x10^6^ freshly isolated NK cells were resuspended in 100 µL of Resuspension Buffer T (Thermo Fisher Scientific) containing 120 µg/mL of endotoxin-free mKeima plasmid. Cells were collected with the Neon pipette and inserted into the pipette station filled with Electrolytic Buffer E2 (Thermo Fisher Scientific). Cells were electroporated with a pulse of 2050V for 20 ms, followed by a second pulse of 500V for 100 ms. Cells were then plated in 6-well plates at 0.5 x 10^6^ cells/mL with pre-warmed RPMI 1640 medium supplemented with GlutaMAX (Gibco), 10% heat-inactivated FBS (HyClone), 1% non-essential amino acids (Gibco) and 1% Sodium Pyruvate (Gibco). Cells were stimulated for 16–18 hours with 10 ng/mL rhIL-12 (Miltenyi Biotec), 100 ng/mL rhIL-15 (Miltenyi Biotec) and 50 ng/mL rhIL-18 (MBL International Corporation), or cultured in media alone for the same time, in the presence of 10 µM CCCP (Sigma-Aldrich) or an equivalent volume of vehicle control (DMSO).

### Statistical analysis and data representation

GraphPad Prism v.8.4 software was used for graphical representation and statistical analysis. FlowJo v10.0.7 software was also used for graphical representation and flow cytometry analyses. Data were represented as the mean ± standard deviation (SD). Grubbs’ test (α = 0.05) was applied to find outliers in the TOM20/cytochrome c co-localization results. Wilcoxon matched-pairs signed rank test or Mann-Whitney test were used to determine significant differences. ns: non-significant, *p < 0.05, **p < 0.01, ****p < 0.0001.

## Results

We have previously described that mitochondrial mass progressively increases following cytokine withdrawal in IL-12/15/18-stimulated NK cells. Despite this increased mitochondrial content, control and CIML NK cells do not show differences in oxidative phosphorylation activity and mitochondrial spare respiratory capacity (18). These results prompted us to hypothesize that following IL-12/15/18 stimulation NK cells could accumulate dysfunctional mitochondria. This effect may have an impact on NK cell survival and function, ultimately hampering the success of CIML NK cell-based therapies.

**Figure 1:**
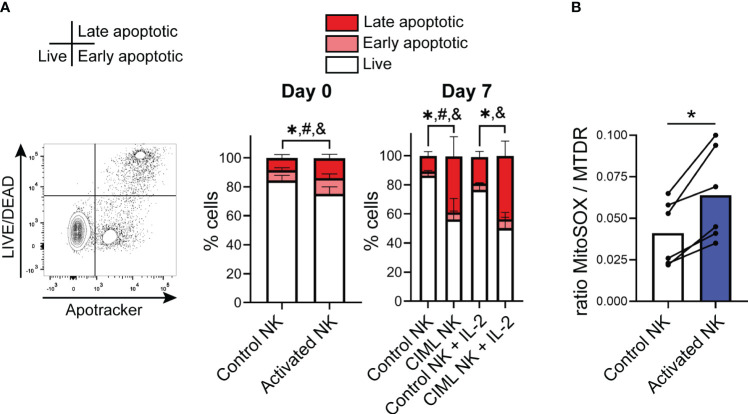
IL-12/15/18-induced cell death in human NK cells and mitochondrial superoxide levels. **(A)** Viability of IL-12/15/18-stimulated NK cells (activated NK) measured by flow cytometry immediately after cytokine stimulation (day 0; n=10) or after culturing them for seven days with IL-15 or IL-2 (day 7; n=8). Live cells (L/D-Apotracker-), early apoptotic cells (L/D-Apotracker+) and late apoptotic (L/D+Apotracker+) are represented with white, light red and dark red colors, respectively. Statistically significant differences (p<0.05) between control and activated NK cells in live, early apoptotic and late apoptotic cells are represented with *, # and &, respectively. **(B)** Mitochondrial superoxide levels upon IL-12/15/18 stimulation measured by flow cytometry using the fluorescent probe MitoSOX normalized to mitochondrial mass and measured with MitoTracker Deep Red (MTDR) (n=6). Significant differences were determined with Wilcoxon matched-pairs signed rank test (*p<0.05).

To understand the effect of cytokine stimulation on NK cell viability and mitochondrial dynamics, we isolated NK cells from healthy donors and measured the viability of IL-12/15/18-stimulated NK cells for 16-18 hours (activated NK) and after culturing them for additional seven days (CIML NK) in the presence of low doses of IL-15 (1 ng/mL) or IL-2 (20 U/mL) in cRPMI media (**Supplementary Figure S1**). In agreement with other authors (19), the viability of IL-12/15/18-stimulated (activated) NK cells was reduced compared with unstimulated (control) NK cells, especially at day 7 (**Figure 1A**). Accordingly, proapoptotic cytochrome c release from mitochondria at day 7 was higher in CIML NK cells (**Supplementary Figure S2**). The impact in NK cell viability of IL-12/15/18 preactivation was further studied culturing the cells in serum-free conditions using NK MACS media. Again, the viability of activated NK cells was reduced in comparison to control NK cells, as it was observed when using serum containing cRPMI media (**Supplementary Figure S3**). Altogether, our data confirmed that stimulation with IL-12/15/18 reduces the viability of NK cells.

Given that mitochondria are crucial organelles in cell death, we next decided to characterize mitochondrial function in CIML NK cells. We found that upon IL-12/15/18 stimulation, NK cells showed increased levels of superoxide accumulation, measured with MitoSOX and normalized to mitochondrial mass (**Figure 1B**, **Supplementary Figure S4**). On the other hand, while Surace et al. have previously described an increase in mitochondrial membrane potential (Δψm) in the CD56^bright^ and a decrease in the CD56^dim^ NK cell subsets upon IL-12/15/18 stimulation (9), in our experiments we did not see significant alterations in the Δψm following cytokine stimulation (**Supplementary Figure S5**). Overall, our findings indicate that IL-12/15/18 stimulation induces an increment in superoxide levels, which may cause oxidative damage and ultimately induce apoptosis.

**Figure 2:**
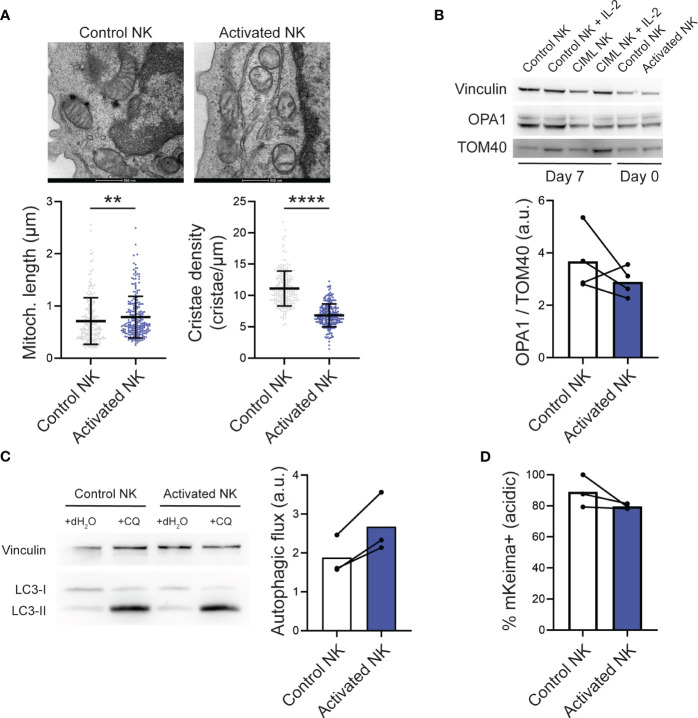
IL-12/15/18-stimulated human NK cells and mitochondrial dynamics. **(A)** Representative electron microscopy micrographs (top) and morphometric analyses of mitochondrial length and cristae density (bottom). Each dot represents a mitochondria using NK cells from 3 different experiments. Scale bar: 500 nm. **(B)** Representative Western Blots of total cell lysates from control and activated NK cells stimulated as indicated (top) and densitometric analyses of OPA1 levels normalized to the mitochondrial protein TOM40 (n=4). Vinculin was used as a loading control. **(C)** Representative Western Blots of total cell lysates from control and CIML NK cells treated as indicated (left) and autophagic flux determined by the densitometric quantification of LC3-II levels in experiments as in the left panel (right; n=3). Autophagic flux was calculated by subtracting the LC3-II expression in cells exposed to vehicle control (distilled water) from the LC3-II expression in cells exposed to chloroquine. **(D)** Mitophagic activity determined by flow cytometry in NK cells transfected with mKeima (n=3). Bars represent mean. a.u., arbitrary units; CQ, chloroquine. Significant differences were determined with Wilcoxon matched-pairs signed rank test, or Mann-Whitney test for panel **(A)** (**p<0.01, ****p<0.0001).

During apoptosis, mitochondria fragment and undergo remodeling of their inner membrane, a process called cristae remodeling that is required for complete cytochrome c release (1). We therefore studied the mitochondrial ultrastructure of NK cells by transmission electron microscopy. Following IL-12/15/18 stimulation (day 0), mitochondria appeared slightly elongated, but more importantly, mitochondrial cristae density significantly decreased (**Figure 2A**). Considering that protein optic atrophy type 1 (OPA1) controls cristae architecture and apoptotic remodeling (1), we measured its levels. Upon IL-12/15/18 stimulation, OPA1 levels tended to be reduced (**Figure 2B**), suggesting that the observed cristae remodeling in stimulated NK cells may be mediated by the decrease in OPA1 levels. Of note, we did not detect clear differences in the mitochondrial morphology of control and CIML NK cells at day 7 (**Supplementary Figure S6**).

In T cells undergoing activation-induced cell death, autophagy is inhibited, promoting an accumulation of dysfunctional mitochondria that ultimately drive T cells to apoptosis (20). At first, we thought that, similar to T cells, IL-12/15/18-induced cell death in NK cells could be due to an impaired removal of dysfunctional mitochondria. Autophagic flux, determined by measuring the autophagic marker LC3-II in basal conditions and after chloroquine inhibition of lysosome acidification and fusion with autophagosomes, was increased upon IL-12/15/18 stimulation (**Figure 2C**). Therefore, we analyzed mitophagy (a form of selective mitochondrial autophagy), which remained unchanged or even lower in activated NK cells compared to control NK cells (**Figure 2D**). Mitophagic activity was measured using the specific genetically encoded probe mKeima (17), a coral-derived pH-dependent fluorescent protein Keima modified with a mitochondria-targeting sequence from COX8. Freshly isolated NK cells were transfected with mKeima and stimulated with IL-12/15/18 for 16-18 hours. Of note, mKeima probe could detect mitophagy changes with the same fidelity in control and stimulated NK cells, as demonstrated by its responsiveness to the well-described inducer of mitophagy CCCP (**Supplementary Figure S7**).

## Discussion

CIML NK cells have become a potent tool in cancer immunotherapy (11–13). The increasing number of clinical trials in which CIML NK cells are being tested and the promising results that they are obtaining highlights their relevance. Yet, these strategies can be further improved, and understanding the features of IL-12/15/18-stimulated NK cells is crucial for this aim. Previously, we hypothesized that CIML NK cells could be accumulating dysfunctional mitochondria, because IL-12/15/18-stimulated NK cells progressively increase mitochondrial mass but there is not a proportional increment of mitochondrial activity (18). Here, we have observed that NK cell viability is reduced upon cytokine stimulation, both in serum-free and serum-containing conditions, which is in agreement with reports from other groups (19, 21). However, it is very important to keep in mind that our data are from *in vitro* experiments, and that once CIML NK cells are infused into the patient, the cytokine milieu may be very different. And considering that infused CIML NK cells showed an upregulation of NKp30 (22), it is tempting to speculate that their prolonged survival may be due to signaling through this receptor, a phenomenon that has been recently described in IL-12-stimulated NK cells (23).

Given the close relationship between mitochondrial fitness and cell death, we conducted a preliminary study to have an overview of mitochondrial dynamics of IL-12/15/18-stimulated NK cells. Our data suggested that NK cells increase mtROS upon cytokine stimulation, which could be indicative of a lower mitochondrial fitness. Considering that a ROS scavenging strategy has improved the viability of NK cells in other models, it could be very interesting to stimulate NK cells in the presence of ROS scavengers such as Tiron or MitoTEMPO (24) and to perform cytokine stimulation in human plasma-like medium (HPLM), which has been described to induce lower amounts of mtROS (25).

We then examined the Δψm and, contrary to what we expected, our results suggested that IL-12/15/18-stimulated NK cells do not show a substantial depolarization. Surace et al. have reported that CD56^bright^ and CD56^dim^ NK cell subsets increase and decrease, respectively, their Δψm upon IL-12/15/18 stimulation, suggesting that distinct subsets can have a different outcome (9). However, our results indicate that changes in the Δψm of CD56^bright^ and CD56^dim^ NK cell subsets are smaller, suggesting that cytokine stimulation does not induce a significant depolarization (data not shown). In addition, we have also studied the ultrastructural morphology of mitochondria and our results show that mitochondrial cristae density is reduced in NK cells upon IL-12/15/18 stimulation, which could be due to a downmodulation of OPA1 expression. We have also analyzed the capacity of IL-12/15/18-stimulated NK cells to degrade dysfunctional mitochondria by measuring autophagic and mitophagic activities. Interestingly, our data suggested that cytokine stimulation induces an increase of autophagy but not mitophagy in human NK cells. Given that a correct elimination of dysfunctional mitochondria through mitophagy is essential for NK cell survival (8), it could be possible that a failure in upregulating mitophagy upon IL-12/15/18 stimulation could explain the accumulation of defective mitochondria and induce subsequent cell death. Our data are preliminary and further research is required to confirm or reject this hypothesis. Nonetheless, it would be worthwhile to study the effect of promoting mitophagy in IL-12/15/18-stimulated NK cells with pharmacological inducers that do not reduce mitochondrial fitness or interfere with metabolic activity (26, 27).

Collectively, our results suggest that upon IL-12/15/18 stimulation, mitophagy is slightly impaired despite the increase in autophagic flux in NK cells. This reduced mitophagy could account for the accumulation of dysfunctional, remodeled mitochondria found in CIML NK cells, ultimately leading to their reduced viability. Our findings shed light on the poorly explored field of mitochondrial dynamics of IL-12/15/18-stimulated NK cells and pave the way to explore new therapeutic strategies focused towards improving mitochondrial fitness of CIML NK cells with the aim of improving their therapeutic efficacy.

## Data availability statement

The raw data supporting the conclusions of this article will be made available by the authors, without undue reservation.

## Ethics statement

The studies involving human participants were reviewed and approved by the Basque Ethics Committee for Clinical Research and the Ethics Committee from the University Hospital of Padova. The patients/participants provided their written informed consent to participate in this study.

## Author contributions

IT and FB conceived the project. IT, LS and FB designed the experiments and analyzed data. IT and VS performed the experiments. IT and FB wrote the manuscript; AA-I, VS, AL-P, AS, GA-P, LA, OZ and LS proofread and edited the manuscript. All authors provided intellectual input and approved the submitted version.
