# Supplementary material for: IL-12/15/18-induced cell death and mitochondrial dynamics of human NK cells
Source: Front Immunol. 2023 Jul 27;14:1211839. doi: 10.3389/fimmu.2023.1211839 (PMC10413107; doi:10.3389/fimmu.2023.1211839)

Supplementary Material for:

IL-12/15/18-induced cell death and mitochondrial dynamics of human NK cells

Iñigo Terrén[1], Víctor Sandá[1], Ainhoa Amarilla-Irusta[1], Ainara Lopez-Pardo[1], Arrate Sevilla[1,2], Gabirel Astarloa-Pando[1], Laura Amo[1,3], Olatz Zenarruzabeitia[1], Luca Scorrano[4,5], Francisco Borrego[1,3*]

[1]Immunopathology Group, Biocruces Bizkaia Health Research Institute, Barakaldo, Spain.
[2]Department of Genetics, Physical Anthropology and Animal Physiology, University of the Basque Country, Leioa, Spain
[3]Ikerbasque, Basque Foundation for Science, Bilbao, Spain.
[4]Department of Biology, University of Padova, Padova, Italy
[5]Veneto Institute of Molecular Medicine, Padova, Italy

*Correspondence: Francisco Borrego MD, PhD
francisco.borregorabasco@osakidetza.eus

This file includes:

- Supplementary Figure S1. Schematic representation of experimental design.
- Supplementary Figure S2. Cytochrome c release of cytokine-induced memory-like (CIML) NK cells at Day 7.
- Supplementary Figure S3. IL-12/15/18-induced cell death in human NK cells cultured in serum-free NK MACS media.
- Supplementary Figure S4. Mitochondrial ROS and mass of IL-12/15/18-stimulated NK cells.
- Supplementary Figure S5. Mitochondrial membrane potential of IL-12/15/18-stimulated NK cells.
- Supplementary Figure S6. Mitochondrial ultrastructure of CIML NK cells.
- Supplementary Figure S7. Gating strategy of mKeima-expressing NK cells.
- Unprocessed original image of western blot from Figure 2B.
- Unprocessed original image of western blot from Figure 2C.

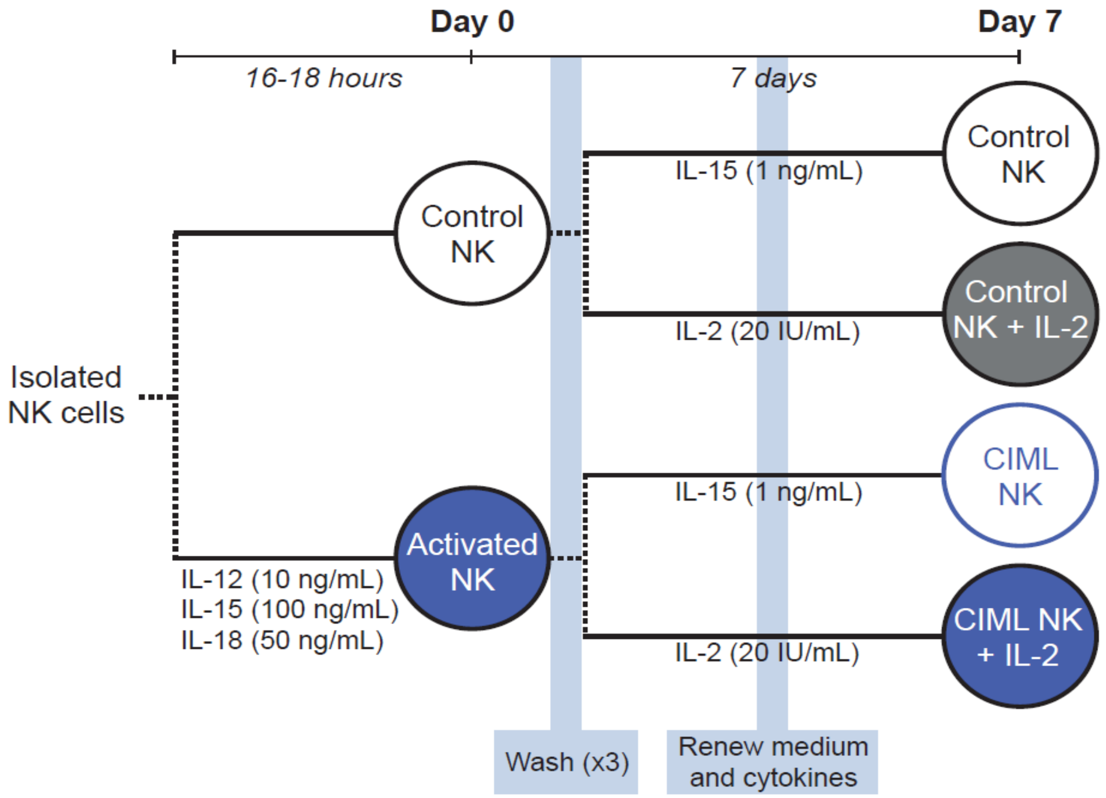

Supplementary Figure S1. Schematic representation of experimental design. Purified NK cells were cultured for 16-18 hours with media alone (control NK) or with a mixture of IL-12, IL-15 and IL-18 (10, 100 and 50 ng/mL, respectively) (activated NK). At this time point (Day 0), cells were collected, washed and analyzed. Additionally, cells were further cultured for 7 days in media containing 1 ng/mL IL-15 (CIML NK) or 20 U/mL IL-2 (CIML NK + IL-2). Media and cytokines were renewed four days after Day 0. After seven days (Day 7), cells were collected, washed and analyzed.

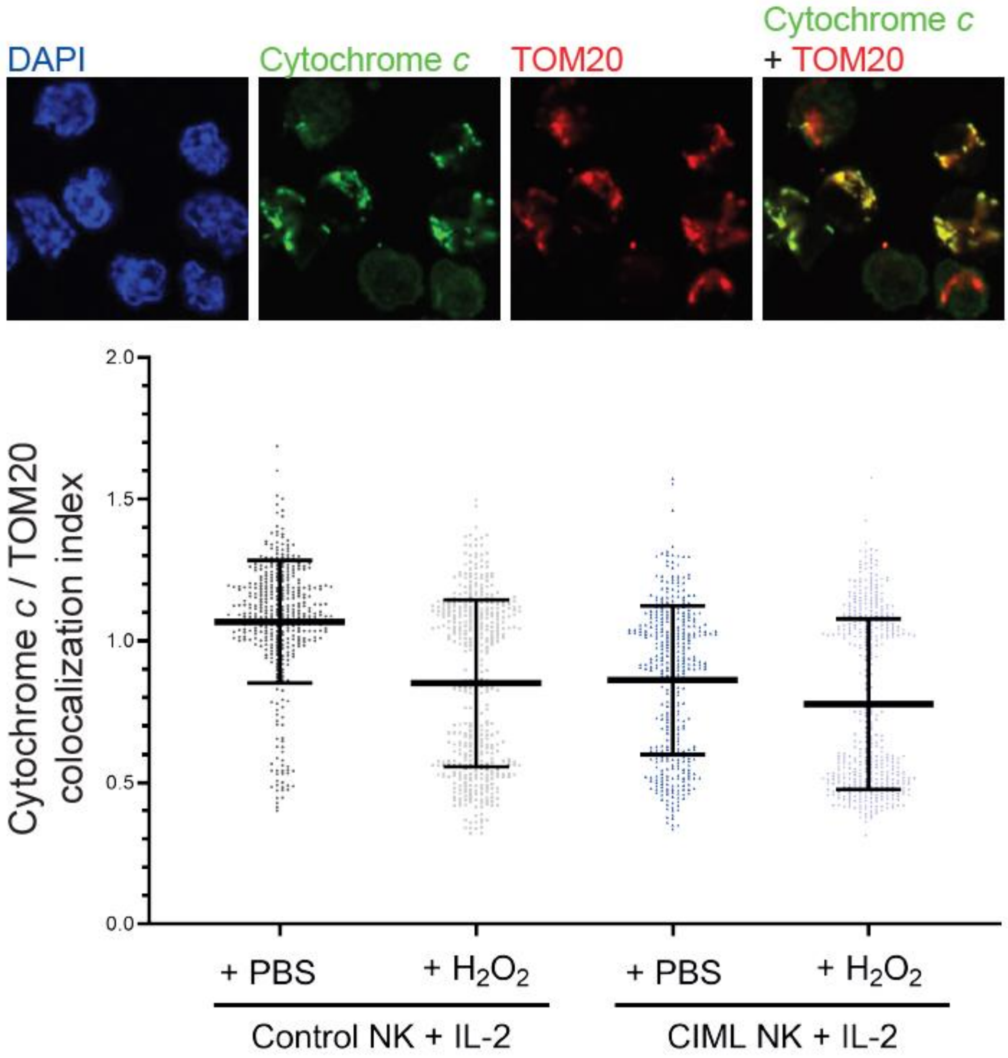

Supplementary Figure S2. Cytochrome c release of cytokine-induced memory-like (CIML) NK cells at Day 7. Control and CIML NK cells cultured with IL-2 for 7 days were washed and incubated for 90 minutes in the presence or absence of 1 mM H_2O_2. Cells were then stained to detect nuclei (DAPI, blue), cytochrome c (green) and mitochondrial protein TOM20 (red). Images show a representative experiment in which cytochrome c can be localized into mitochondria or cytosol in different cells. Graph shows TOM20 and cytochrome c co-localization index. Means ± SD are depicted. Grubbs' test ($\alpha = 0.05$) was applied to exclude outliers. Each dot represents one analyzed cell from a total of 400 to 470 cells (n=1).

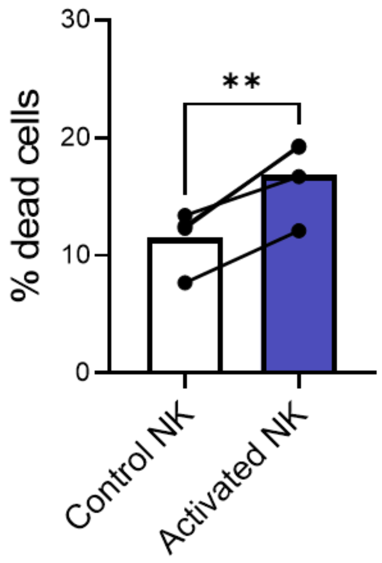

Supplementary Figure S3. IL-12/15/18-induced cell death in human NK cells cultured in serum-free NK MACS media. Viability of IL-12/15/18-stimulated NK cells (activated NK), cultivated in serum-free NK MACS media, measured by flow cytometry immediately after stimulation (Day 0; n=4). Data represent the percentage of dead cells, identified as LIVE/DEAD+ cells. Bars represent the means. Each dot represents an independent experiment from a different donor. Significant differences were determined with paired t-test test (**p<0.01).

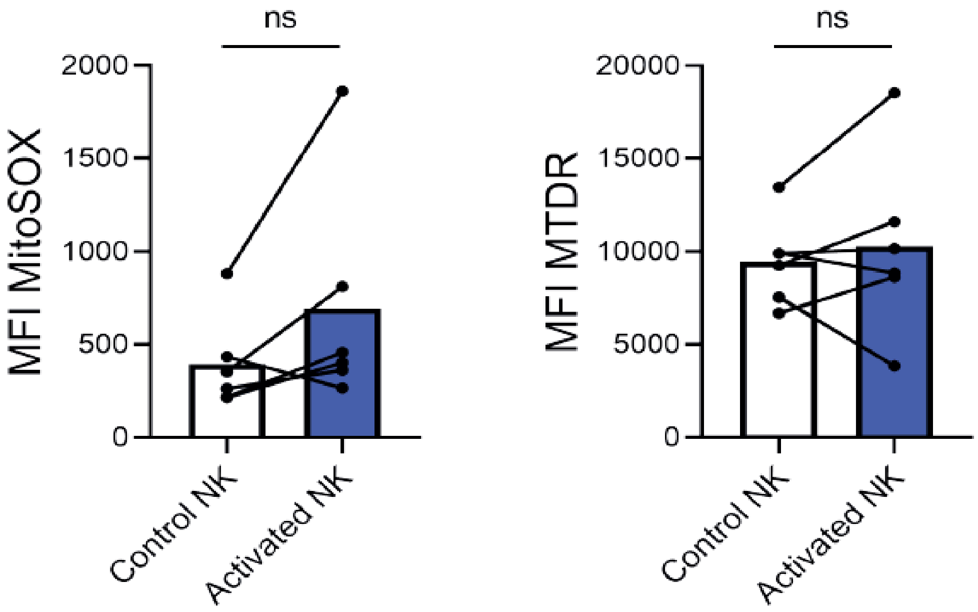

Supplementary Figure S4. Mitochondrial ROS and mass of IL-12/15/18-stimulated NK cells. Bar graphs show levels of mitochondrial ROS (mtROS), measured as the median fluorescence intensity (MFI) of MitoSOX, and mitochondrial mass, measured as the MFI of MitoTracker Deep Red (MTDR), of control NK cells and IL-12/15/18-stimulated NK cells (activated NK) at Day 0. Bars represent the means. Each dot represents an independent experiment from a different donor (n=6). Significant differences were determined with Wilcoxon matched-pairs signed rank test.

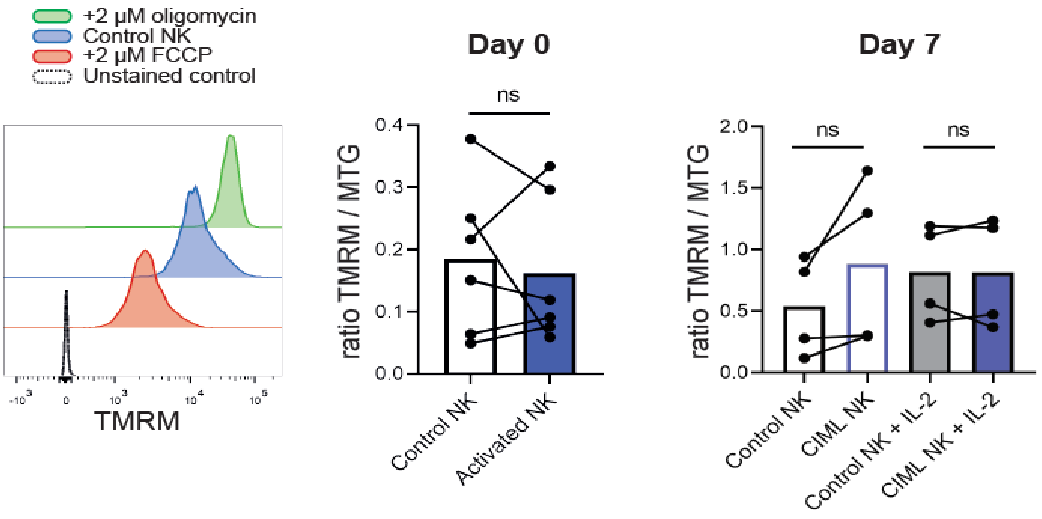

Supplementary Figure S5. Mitochondrial membrane potential of IL-12/15/18-stimulated NK cells. Histograms show a representative example of tetramethylrhodamine (TMRM) fluorescence of unstained NK cells and NK cells cultured with or without 2 µM FCCP or 2 µM oligomycin. Bar graphs show the mitochondrial membrane potential (ΔΨm) measured as the ratio between the MFI of TMRM and MitoTracker Green (MTG) of control NK cells and IL-12/15/18-stimulated NK cells (activated NK) at Day 0, and control and CIML NK cells at Day 7. Bars represent the means. Each dot represents an independent experiment from a different donor (n=6). Significant differences were determined with Wilcoxon matched-pairs signed rank test.

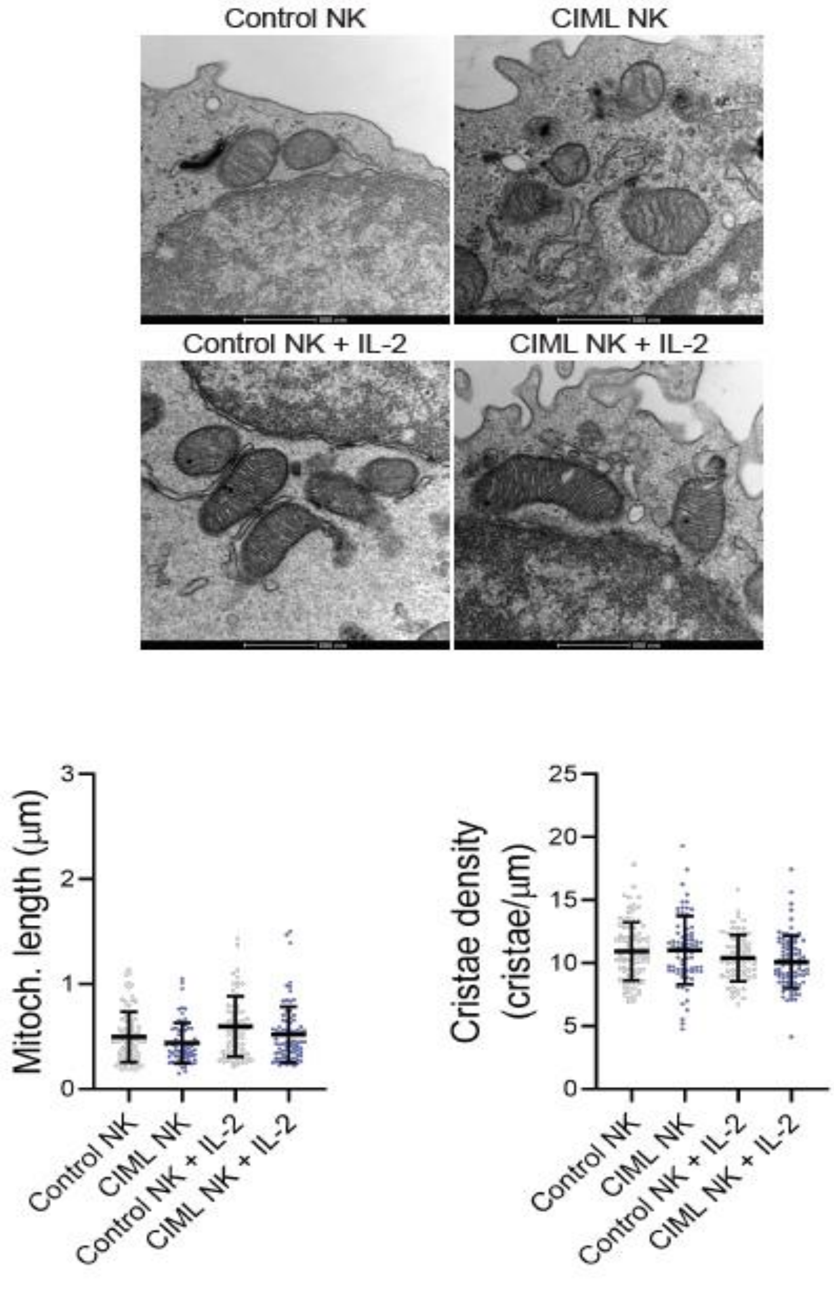

Supplementary Figure S6. Mitochondrial ultrastructure of CIML NK cells. On the top, representative electron micrographs of control and activated NK cells cultured for 7 days with IL-15 (control NK and CIML NK) or IL-2 (control NK + IL-2 and CIML NK + IL-2). Scale bar: 500 nm. On the bottom, bar graphs show mitochondrial length and cristae density measured as the number of cristae divided by the mitochondrial length of each mitochondrion. Bars represent the means ± SD (n=2).

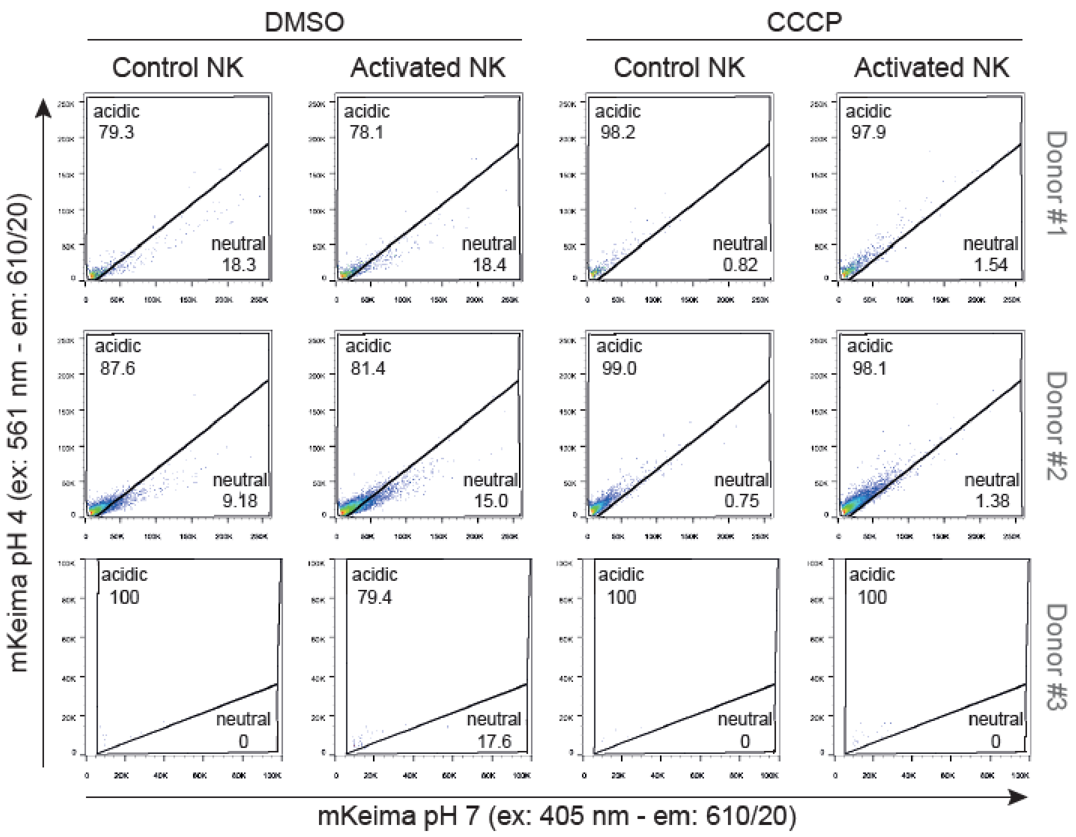

Supplementary Figure S7. Gating strategy of mKeima-expressing NK cells. Freshly isolated NK cells were transfected with mKeima-Red-Mito-7 plasmid using Neon Transfection system. Then, cells were stimulated or not with IL-12/15/18 for 16-18 hours in the presence and absence of 10 µM CCCP or an equivalent volume of DMSO and analyzed by flow cytometry. Pseudocolor plots show the mitophagic activity of control and activated NK cells, measured as the percentage of NK cells expressing mKeima in a neutral pH (mitochondria are located in the cytosol) and mKeima in an acidic pH (mitochondria are located within autolysosomes, i.e., autophagosomes fused to lysosomes). mKeima in neutral pH was excited with the violet laser (405 nm) and detected with a filter of 610/20 nm. mKeima in acidic pH was excited with the yellow-green laser (561 nm) and detected with a filter of 610/20 nm. Gates to discriminate between mKeima in acidic and neutral pH were determined in the cells cultured with the mitophagy-inducer CCCP (n=3).

Unprocessed Image of Figure 2B

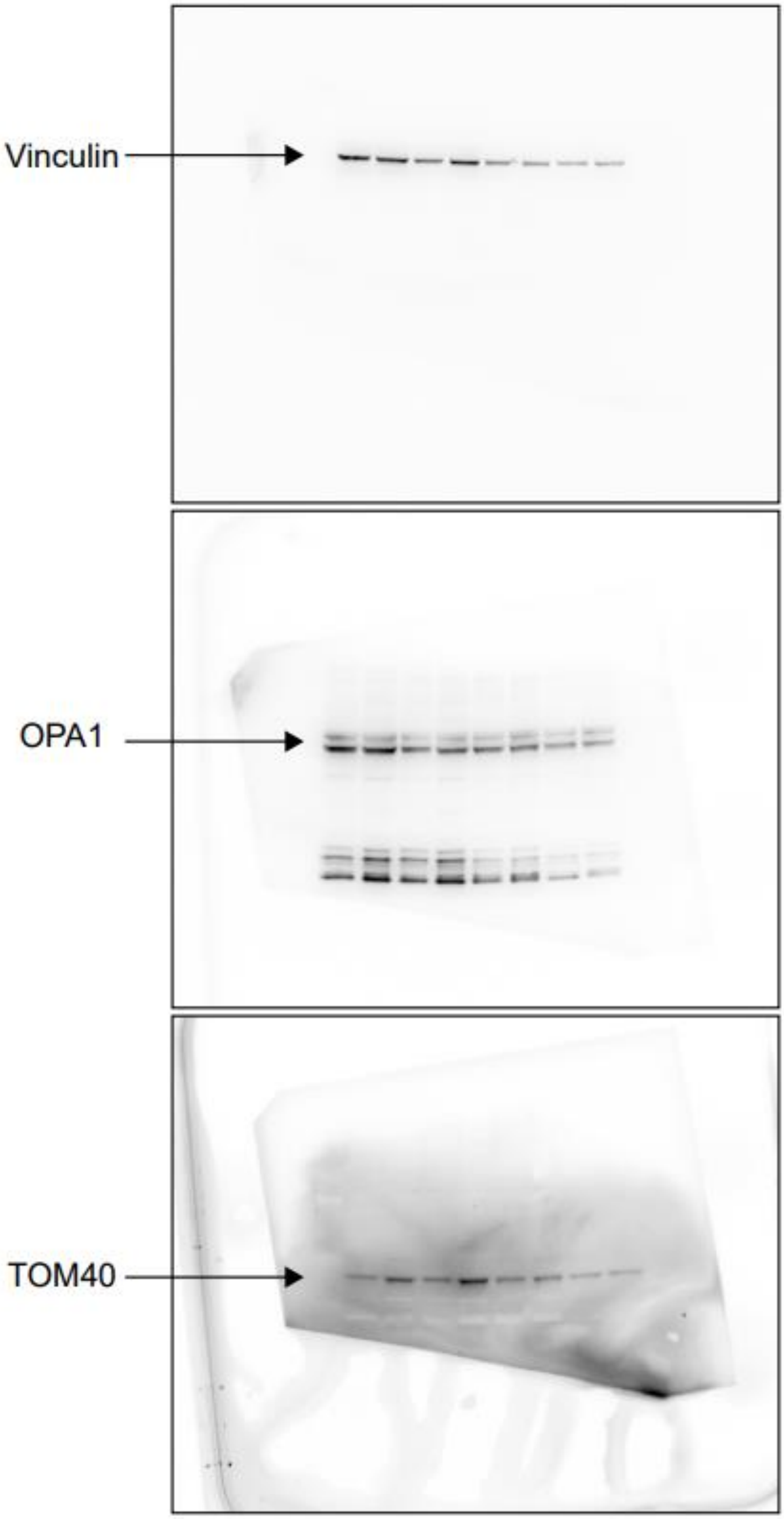

Unprocessed Image of Figure 2C

Vinculin

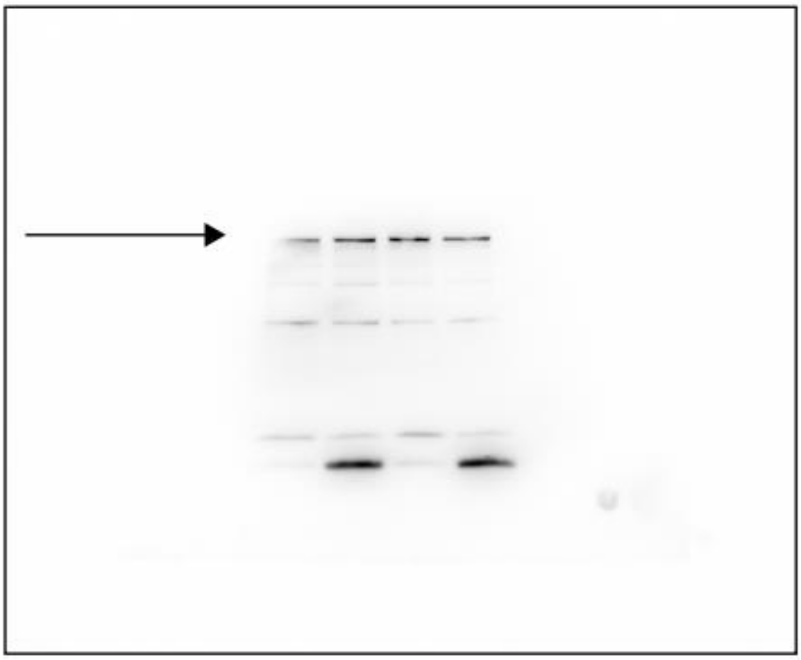

LC3

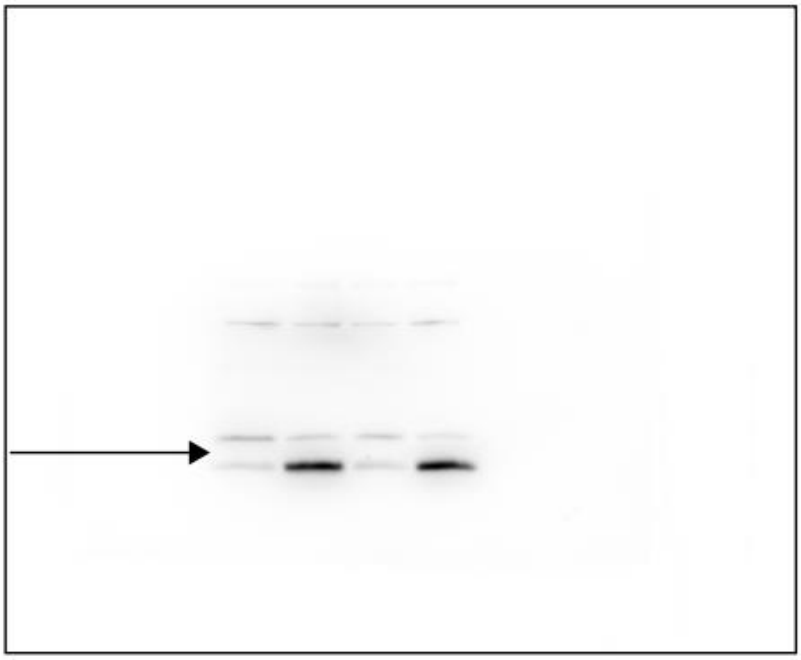

Supplement: Supplementary file 1 [file DataSheet_1.pdf]
